# Mitochondrial gene polymorphism is associated with gut microbial communities in mice

**DOI:** 10.1038/s41598-017-15377-7

**Published:** 2017-11-10

**Authors:** Misa Hirose, Axel Künstner, Paul Schilf, Annika Sünderhauf, Jan Rupp, Olaf Jöhren, Markus Schwaninger, Christian Sina, John F. Baines, Saleh M. Ibrahim

**Affiliations:** 1 Lübeck Institute of Experimental Dermatology, University of Lübeck, Lübeck, Germany; 2 Group for Medical Systems Biology, Lübeck Institute of Experimental Dermatology, University of Lübeck, Lübeck, Germany; 3 Institute of Cardiogenetics, University of Lübeck, Lübeck, Germany; 4 Max Planck Institute for Evolutionary Biology, Evolutionary Genomics, Plön, Germany; 5 Institute of Nutritional Medicine, University of Lübeck, Lübeck, Germany; 6 Department of Infectious Diseases and Microbiology, University of Lübeck, Lübeck, Germany; 7 Center of Brain, Behavior and Metabolism, University of Lübeck, Lübeck, Germany; 8 Institute of Experimental and Clinical Pharmacology and Toxicology, University of Lübeck, Lübeck, Germany; 9 Institute for Experimental Medicine, Evolutionary Genomics, Kiel, Germany; 10 College of Medicine and Sharjah Institute for Medical Research, University of Sharjah, Sharjah, United Arab Emirates

## Abstract

Gut microbial communities are key mediators of health and disease and have the capacity to drive the pathogenesis of diverse complex diseases including metabolic and chronic inflammatory diseases as well as aging. Host genetics is also a major determinant of disease phenotypes, whereby two different genomes play a role, the nuclear (nDNA)- and mitochondrial genome (mtDNA). We investigated the impact of mutations in mtDNA on the gut microbiota using conplastic mouse strains exhibiting distinct mutations in their mtDNA on an identical nDNA. Each of three strain tested harbors a distinct gut microbiota, ranging from differences at the phylum- to operational taxonomic units level. The *C57BL*/*6J-mt* ^*FVB*/*NJ*^ strain, carrying a mutation in the mitochondrial ATP8 synthase gene, exhibits higher Firmicutes abundance than Bacteroidetes, indicating a possible indicative for metabolic dysfunctions. In line with this, the *C57BL*/*6J-mt* ^*FVB*/*NJ*^ displays a variety of different phenotypes, including increased susceptibility to metabolic-related and inflammatory disorders. Furthermore, we discuss the cross-talk between mitochondrial genome/mitochondria and commensal microbiota in relation to clinical phenotypes. In summary, we demonstrate that mutations in mtDNA lead to significant differences in the composition of gut microbial communities in mice. Such differences may facilitate the emergence of metabolic disease and therefore constitute potential therapeutic targets.

## Introduction

In recent years a pivotal role of the host-associated microbiota in the emergence of chronic diseases has become apparent, largely due to the realization that these communities are much more variable among individuals than previously anticipated^1^. Chronic diseases, including metabolic diseases such as obesity and type 2 diabetes (T2D)^2,3^, inflammatory disorders such as inflammatory bowel disease^4^ as well as aging^5^ are in most cases assumed to be driven by a multitude of factors, including the host’s individual traits as well as environmental factors. The relative importance of host genetics, both nuclear DNA (nDNA)^2^ and mitochondrial DNA (mtDNA)^6,7^, on such conditions is undisputed, although it is conceivable that some host genes may modulate diseases via way of their influence on the microbiota.

Mammalian mtDNA is circular, approximately 16 kilobases in length and encodes 13 protein-coding genes, 22 transfer RNA genes and 2 ribosomal RNA genes. Herein, the proteins encoded are part of the complexes I, III, IV and V of the mitochondrial oxidative phosphorylation (OXPHOS) subunits^6,8^. Consequently, mutations in the mtDNA impact OXPHOS functions, including adenosine triphosphate (ATP) production, reactive oxygen species (ROS) production and mitochondrial membrane potential. These all contribute to pivotal cellular activities, such as cell activation, proliferation and cell death^9^.

It is therefore not surprising that mutations in mtDNA can have severe consequences for the entire organism, which is well exemplified by a number of mitochondrially inherited disorders such as Leber’s hereditary optic neuritis (LHON), mitochondrial encephalomyopathy, lactic acidosis, and stroke-like episode (MELAS)^6^.

Mitochondrial mutations are also associated with chronic diseases including T2DM^10^, serum lipid levels^11^, obesity^12^, cardiovascular diseases^13^ and neurodegenerative diseases^14^, as well as autoimmune diseases such as rheumatoid arthritis^15^ and systemic lupus erythematosus (SLE)^16^. Given that these same chronic diseases are also associated with shifts in the gut microbiota^17–22^, it is possible that mtDNA mutations may be linked to such changes.

Ma *et al*. recently reported that certain mitochondrial haplogroups are associated with the relative abundance of *Bacteroides* and *Prevotella*, two prevalent taxa of the gut microbiota^23^. While differences are apparent according to region-defined haplogroups (Europeans, Asian, and Mexican Americans), non-haplogroup defining single nucleotide polymorphism (SNP) could not be clearly associated to variation in microbial communities. This limitation is in part due to the parallel impact of the highly variable nuclear genome on the composition of the gut microbiota^24^, making it difficult to reveal the effect of mitochondrial polymorphism.

This obstacle can be circumvented by the use of conplastic mouse strains, which all share the same nuclear genome but differ in their mtDNA. Since common inbred mouse strains demonstrate unique mitochondrial genomes^25–27^ and the mitochondria are strictly maternally inherited, we systematically generated conplastic strains carrying mostly single mutations in mtDNA with a *C57BL*/*6J* nuclear background by repeatedly backcrossing female mitochondrial-donor strains with male *C57BL*/*6J* mice over 12 generations^27^. This unique resource allowed us to investigate the impact of defined mtDNA mutations on gut microbiota composition in the absence of confounding variation in the nuclear genome. For this purpose, we selected two conplastic strains, *C57BL*/*6J-mt* ^*FVB*/*NJ*^, which carries a mutation (m.7778 G > T) in the mitochondrial ATP synthase 8 gene (*mt-Atp8*) as a representative of a conplastic strain carrying a single mtDNA mutation, and the *C57BL*/*6J-mt* ^*NZB*/*BlnJ*^ strain, which carries multiple mutations in the mtDNA, and compared the composition of their gut microbial communities to those of the native *C57BL*/*6J* strain.

Accordingly, this strategy unveils a number of strain-specific differences in gut bacterial communities with respect to mtDNA mutations. Intriguingly, *C57BL*/*6J-mt* ^*FVB*/*NJ*^, with their single mutation in the *mt-Atp8* gene, display the greatest level of differentiation in their gut microbiota compared to *C57BL*/*6J* and *C57BL*/*6J-mt* ^*NZB*/*BlnJ*^, pointing towards a major role of mitochondrial ATP synthase 8 in shaping the gut microbiota.

## Results

### High strain-dependent diversity of the gut microbiota diverse in conplastic mouse strains

#### Mitochondrial mutations impact on gut bacterial communities at both the phylum and family levels

To evaluate the impact of mtDNA mutations on the gut microbiota, we analyzed 48 individuals from three conplastic mouse strains (*C57BL*/*6J*, *C57BL*/*6J-mt* ^*FVB*/*NJ*^ and *C57BL*/*6J-mt* ^*NZB*/*BlnJ*^, N = 16/strain) using 16S rRNA gene amplicon sequencing (V1–V2 region on the Illumina MiSeq). For each individual, 12,500 contigs were randomly selected and clustering sequences into operational taxonomic units (OTUs) yielded 4,853 OTUs after excluding singletons (see Material and methods).

**Figure 1:**
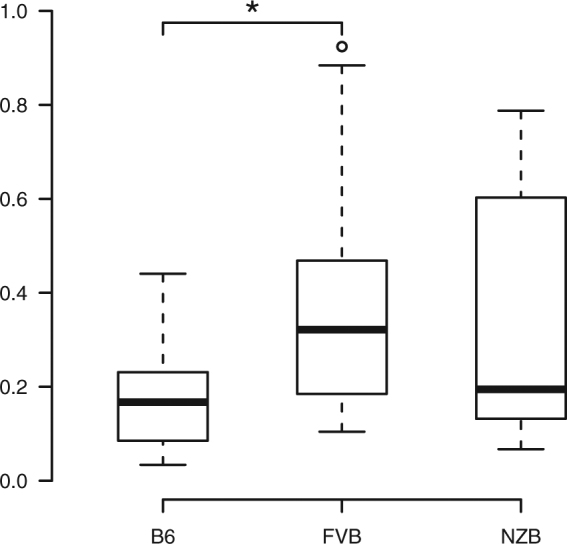
The ratio of Firmicutes and Bacteroidetes significantly differs between conplastic strains. *P = 0.0230 (Kruskal-Wallis test), *P < 0.05 (Dunn test). B6, *C57BL*/*6J*; FVB, *C57BL*/*6J-mt* ^*FVB*/*NJ*^; NZB, *C57BL*/*6J-mt* ^*NZB*/*BlnJ*^.

At the level of phylum abundances, the gut microbiota of conplastic mouse strains was similar to previously described studies of *C57BL*/*6J* mice, with Bacteroidetes being most abundant, followed by Firmicutes and Proteobacteria (Table 1)^28^. However, significant strain-dependent differences were also apparent, characterized by *C57BL*/*6J* mice differing from *C57BL*/*6J-mt* ^*FVB*/*NJ*^ mice in the abundance of Bacteroidetes, Deferribacteres, Firmicutes, Proteobacteria, and Tenericutes (Mann-Whitney *U* test, *P*_adj_ < 0.05) and from *C57BL*/*6J-mt* ^*NZB*/*BlnJ*^ mice in the abundance of Deferribacteres and Tenericutes (*P*_adj_ < 0.05). The *C57BL*/*6J-mt* ^*FVB*/*NJ*^ and *C57BL*/*6J-mt* ^*NZB*/*BlnJ*^ strains on the other hand did not show any significant differences in phylum abundances. *C57BL*/*6J* and *C57BL*/*6J-mt* ^*FVB*/*NJ*^ mice also significantly differed in the ratio of Firmicutes to Bacteroidetes (*P* = 0.0230, Kruskal-Wallis test; Dunn test, *P*_adj_ = 0.0111), which was higher in *C57BL*/*6J-mt* ^*FVB*/*NJ*^ mice than in *C57BL*/*6J* mice (Fig. 1). This finding is intriguing given the association of Firmicutes to Bacteroidetes ratios being associated with obesity^17,29^.

**Table 1:** Taxonomic analysis at the phylum level in conplastic strains.

Phylum	*C57BL*/*6J* (*%*)	*C57BL*/*6J-mt* ^*FVB*/*NJ*^ (*%*)	*C57BL*/*6J-mt* ^*NZB*/*BlnJ*^ (*%*)
Actinobacteria	0.09	0.08	0.16
Bacteroidetes	80.86	66.44*	70.51
Cyanobacteria	0.22	0.26	0.44
Deferribacteres	0.05	0.47*	0.21^#^
Firmicutes	13.11	22.20*	20.43
Proteobacteria	5.32	9.10*	7.81
Tenericutes	0.03	0.27*	0.18^#^
Unclassified	0.19	0.35	0.21
Verrucomicrobia	0.13	0.83	0.04

*P_adj_ < 0.05 (compared with *C57BL*/*6J*); ^#^ *P*_adj_ < 0.05 (compared with *C57BL*/*6J*).

Next, we compared the gut microbiota of the conplastic strains at the family level. *Bacteroidales S24-7* group, *Lachnospiraceae*, and *Helicobacteraceae* were in general the most abundant families (Supplementary Table S1), and there were significant differences in the abundance between the individual conplastic strains. *C57BL*/*6J* mice significantly differed from *C57BL*/*6J-mt* ^*FVB*/*NJ*^ mice in the abundance of *Anaeroplasmataceae*, *Bacteroidales S24-7 group*, *Deferribacteraceae*, *Desulfovibrionaceae* and *Helicobacteraceae* (MWU-test, *P*_adj_ < 0.05) and from *C57BL*/*6J-mt* ^*NZB*/*BlnJ*^ mice in *Anaeroplasmataceae* (*P*_adj_ = 0.0468) (Supplementary Table S1). *C57BL*/*6J-mt* ^*FVB*/*NJ*^ and *C57BL*/*6J-mt* ^*NZB*/*BlnJ*^ mice showed significant differences in the abundance of *Christensenellaceae*, *Desulfovibrionaceae*, and *Family XIII* (*P*_adj_ < 0.05).

#### The impact of mtDNA mutations on alpha diversity and beta diversity

To characterize the level of diversity within strains, alpha diversity was analyzed by applying Mann-Whitney *U* tests for the Chao1, Shannon and Simpson index. None of the three strains significantly differ in alpha diversity (*P*_adj_ > 0.05; Mann-Whitney *U* tests).

Next, to characterize diversity between strains, beta diversity was analyzed using the unweighted UniFrac distance metric, revealing significant differences between strains (Kruskal-Wallis test, *P* = 0.0252). Applying a *post-hoc* test identified significant differences in beta diversity between *C57BL*/*6J* and *C57BL*/*6J-mt* ^*FVB*/*NJ*^ as well as between *C57BL*/*6J-mt* ^*FVB*/*NJ*^ and *C57BL*/*6J-mt* ^*NZB*/*BlnJ*^ mice (Dunn test, *P* = 0.0144 and *P* = 0.0276, respectively). To explore the distance between the communities and the effect of sex on differences in beta diversity, we applied the multivariate analysis of variance (*adonis*) on the UniFrac distance. Using this approach, we discovered a significant difference in beta diversity between strains (*adonis*: *P* = 0.001, R^2^ = 0.1168) and sexes (*P* = 0.001, R^2^ = 0.0376). We also verified an interaction between strain and sex (*P* = 0.002, R^2^ = 0.0614).

**Figure 2:**
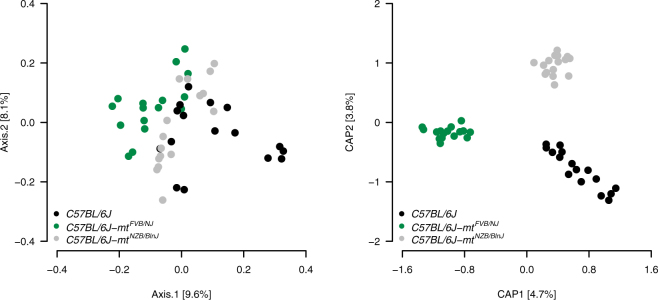
Beta diversity of gut microbiota in conplastic strains. (**A**) Principal coordinate analysis (PCoA) on unweighted UniFrac distance. (**B**) Capscale plot on unweighted UniFrac distance. Strain was taken as constraint, and correction for sex effect was applied. The CAP1 and CAP2 were found to be significant with respect to strain with 999 permutations (*P* = 0.001 for CAP1 and CAP2).

To further detail the differences in the microbiota of the three strains, we conducted a principle coordinate analysis (PCoA). The microbiota of the strains is distinguishable along the first principle coordinate, which explains 9.6% of the variance (Fig. 2A). To investigate this pattern further, we applied a constrained analysis of principal coordinates on the data with strain as an explanatory variable with a correction for sex effects, which reveals a highly significant effect (ANOVA: *P* = 0.001, F_2,44_ = 2.030) with R^2^ = 0.0819 (R^2^_adj_ = 0.0425) (Fig. 2B).

#### Specific gut microbiota are associated with conplastic mouse strains

To identify bacterial species-level OTUs that are significantly associated with a specific cohort, we applied indicator species analysis using the method of Dufrêne and Legendre^30^. This revealed four OTUs associated with *C57BL*/*6J*, 14 with *C57BL*/*6J-mt* ^*FVB*/*NJ*^ and two with the *C57BL*/*6J-mt* ^*NZB*/*BlnJ*^ strain (Supplementary Table S2).

### Basal phenotypes of conplastic strains carrying a mutation in *mt-Atp8*

Given the marked differences of microbiota between *C57BL*/*6J-mt* ^*FVB*/*NJ*^ and the two other mouse strains, we next characterized *C57BL*/*6J-mt* ^*FVB*/*NJ*^ mice, which carry a mutation in the mitochondrially-encoded ATP synthase 8 gene (*mt-Atp8*), with respect to their general and cellular metabolic phenotypes.

Using a separate cohort of mice than those included in the gut microbiota analysis, we first weighed *C57BL*/*6J-mt* ^*FVB*/*NJ*^ and *C57BL*/*6J* mice at 3, 6, 12, 18 and 24 months of age. In males of both strains body weight increased with age until 18 months and declined without any significant difference between the two strains (Supplementary Fig. S1,A). In contrast, in females body weight increased with time throughout the observation period of 24 months. While there was no difference between the two strains at younger ages, starting with 12 months *C57BL*/*6J-mt* ^*FVB*/*NJ*^ females were significantly lighter than *C57BL*/*6J* females (two-way ANOVA, *P* = 0.0279, *P* = 0.0284, and *P* < 0.0001, respectively, Supplementary Fig. S1,B).

By indirect calorimetric cage system, we assessed the basal metabolism of *C57BL*/*6J-mt* ^*FVB*/*NJ*^ and *C57BL*/*6J* mice at the age of 3 months. While the two strains displayed an equal respiratory exchange ratio (RER), energy expenditure and food and water intake, *C57BL*/*6J-mt* ^*FVB*/*NJ*^ mice were significantly less active than *C57BL*/*6J* mice, indicating an enhanced metabolic base level rate in the *C57BL*/*6J-mt* ^*FVB*/*NJ*^ strain (*P* = 0.0331, *t* test, Supplementary Fig. S1,C–G).

**Figure 3:**
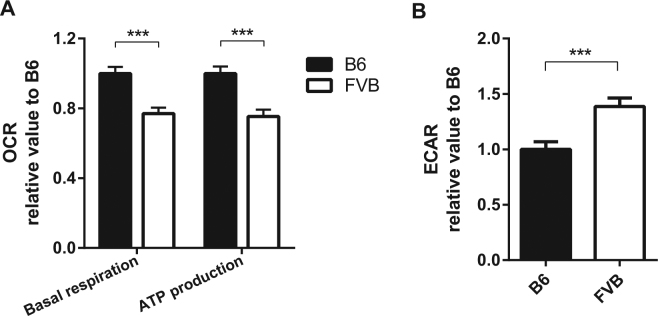
Reduced oxygen consumption levels and Increased levels of glycolysis in primary lymphocytes isolated from *C57BL*/*6J-mt* ^*FVB*/*NJ*^ compared with cells from *C57BL*/*6J*. (**A**) The oxygen consumption ratio (OCR) was measured in the primary lymphocytes from peripheral lymph nodes. Basal respiration and ATP production levels were significantly less in *C57BL*/*6J-mt* ^*FVB*/*NJ*^ compared to *C57BL*/*6J*. (**B**) The extracellular acidification rate (ECAR), which indicates glycolysis levels, in the primary lymphocytes was measured using a Seahorse XF analyzer. Data presented in the graph was normalized with the value of *C57BL*/*6J*. B6: C57BL/*6J*, FVB: *C57BL*/*6J-mt* ^*FVB*/*NJ*^. ***P < 0.001, *t* test.

To evaluate the mitochondrially-related metabolic status, we measured the oxygen consumption and glycolysis levels in primary lymphocytes of *C57BL*/*6J-mt* ^*FVB*/*NJ*^ and *C57BL*/*6J* mice in a cell metabolism analyzer (Seahorse XF analyzer). *C57BL*/*6J-mt* ^*FVB*/*NJ*^ lymphocytes consumed significantly less oxygen than their *C57BL*/*6J* counterparts (Fig. 3A), but instead displayed higher levels of glycolysis (*P* = 0.0016, Fig. 3B). These data indicate that the mutation in *mt-Atp8* impairs mitochondrial oxidative phosphorylation and consequently reduces ATP production, which is in turn compensated by an enhanced level of glycolysis.

Notably, there was no difference in the overall lifespan of *C57BL*/*6J-mt* ^*FVB*/*NJ*^ and *C57BL*/*6J* mice under normal housing condition (Supplementary Fig. S2,A–C, and Supplementary Table S3).

## Discussion

In this study, we provide the first direct evidence that mutations in the mitochondrial genes significantly impact the composition of the gut microbial community in mice. Among several mitochondrial genes participating in this regulation, the *mt-Atp8* appears to be of particular importance, as the mutation (a single mutation, m.7778 G > T) in this gene alone leads to notable differences in gut microbial composition compared to *C57BL*/*6J* mice.

The link between mitochondria and commensal bacterial composition, particularly in the gut, is increasingly receiving attention^31,32^. Mitochondria are the cellular organelles that function as metabolic centers, controlling energy production by OXPHOS respiration, glycolysis and lipid metabolism. In the host cell metabolism, mitochondria utilize metabolites produced by the intestinal commensal bacteria, such as acetyl coenzyme A (acetyl-CoA) and fuel mitochondria to produce cellular energy in the form of ATP^9^. In addition to cellular bioenergetic functioning, mitochondria generate ROS during OXPHOS respiration, which plays a role in oxidative stress to damage cells, apoptosis and inflammatory signal transduction, in addition to being bactericidal^33,34^. Thus, mitochondria appear to be capable of influencing gut microbiota composition in multiple ways.

The communication of gut microbiota to mitochondria can be outlined as follows: Anaerobic bacteria ferment undigested dietary components such as dietary fibers and produce gases and organic acids, especially three short chain fatty acids (SCFAs): acetate, propionate and butyrate^35^. In addition, secondary bile acids such as lithocholic acids and deoxycholic acids are also produced by bacteria residing in the gut^36^. Regarding SCFAs, butyrate and propionate are known to inhibit the activity of histone deacetylases (HDACs) in colon cells and immune cells, particularly regulatory T cells^37,38^, accordingly increasing the hyperacetylation of histones, followed by controlling gene transcription involved in signal transduction such as *FOXP3*, *NFκB*, *SIRT1* and *PPARγ* ^35^, which influence the inflammatory response and mitochondrial functioning. Acetate as well as butyrate are converted into acetyl-CoA and tricarboxylic acid (TCA) cycle intermediates in mitochondria, which are used in various bioenergetics processes^39^. Secondary bile acids interact with mitochondria by modulating transcription factors that relate to lipid and carbohydrate metabolism^40^, thus, these microbial products act as signaling molecules that modulate mitochondrial bioenergetic functioning. Recently, it was found that a shift in intestinal microbiota correlated with expression of a panel of mitochondrial proteins in Crohn´s disease patients, indicating that cross-talk between the microbiota and host mitochondria may be an important factor in disease pathology^41^.

Bacterial taxa whose abundance varies according to the conplastic strains investigated represent candidates for involvement in mitochondrial bioenergetic functions. At the phylum level, the most prominent differences in taxon abundance relative to *C57BL*/*6J* were observed in *C57BL*/*6J-mt* ^*FVB*/*NJ*^, whereby a total of four phyla including the two major phyla Bacteroidetes and Firmicutes displayed significant differences. Moreover, the ratio of Firmicutes to Bacteroidetes, which reportedly correlates with metabolic phenotypes such as obesity^17,29^, was significantly increased in *C57BL*/*6J-mt* ^*FVB*/*NJ*^. Thus, below we discuss taxon abundance differences with respect to *C57BL*/*6J-mt* ^*FVB*/*NJ*^ in particular.

Bacteroidetes and Firmicutes are the two most dominant bacterial phyla of the gut microbiota^35^. In line with analysis at the phylum level, the abundance of *Bacteroidales S27-4 subgroup* (belonging to Bacteroidetes) is significantly reduced in *C57BL*/*6J-mt* ^*FVB*/*NJ*^. This family was previously shown to fluctuate in abundance in several mouse studies, including increased abundance in mice fed with a high-fat diet^42^, and mice in remission of colitis after treatment^43^. Thus, alterations in abundance of *Bacteroidales S27-4* may reflect the metabolic status of the host. Further, OTUs belonging to *Alistipes* are among the indicator species of *C57BL6-mt* ^*FVB*/*NJ*^, and reported to be increased in high-fat diet fed mice^42^. *Alistipes* abundance negatively correlates with D-pinitol, which is converted to myo-inositol and further processed to acetyl-CoA^44^, a key metabolite to fuel mitochondrial metabolism.

Other taxa associated to *C57BL6-mt* ^*FVB*/*NJ*^ belong to the Firmicutes, such as *Lachnospiraceae spp*. Members of this family are generally thought to exert anti-inflammatory effects through the biosynthesis of butyrate, whereas others have also been associated with increased disease susceptibility, including to lupus^45^ and diabetes^46^. These contrasting aspects may be due to differences in SCFA production among individual members of *Lachnospiraceae* ^47^. For example, some species convert lactate to propionate via the acrylate pathway^48^. Propionate is thought to regulate lipid synthesis in the liver^49^. Given that *C57BL*/*6J-* ^*mtFVB*/*NJ*^ is susceptible to many metabolic related disorders, it is conceivable that these bacteria may impact metabolic phenotypes in this strain.

Interestingly, *Desulfovibrionaceae* (Proteobacteria) are among the taxa exhibiting increased abundance in *C57BL*/*6J-mt* ^*FVB*/*NJ*^. This family is of particular interest due to their capacity to produce hydrogen sulfide (H_2_S)^50^. Sulfate-reducing bacteria-derived H_2_S reportedly contribute to T cell activation and proliferation^51^, suggesting a contribution of *Desulfobibrionaeceae* to inflammatory phenotypes. Nevertheless, both pro-inflammatory and anti-inflammatory effects of sulfate-reducing bacteria have been reported^52^. Importantly, however, it was also reported that H_2_S regulates mitochondrial bioenergetic functioning, particularly on mitochondrial complex IV^53^.

Our phenotyping of *C57BL*/*6J-mt* ^*FVB*/*NJ*^ mice demonstrates a skewed cellular metabolism, i.e. reduction in OXPHOS respiration and increased glycolysis, which could alter cellular energy levels in the gut as well as in immune cells. In addition, it could lead to differences in metabolite profiles, including SCFA, which we have previously reported^54^. Changes in the host metabolic profile could accordingly impact other aspects of bacterial community structure in the gut, leading to further imbalances and potential disease susceptibility. On the other hand, *C57BL*/*6J-mt* ^*FVB*/*NJ*^ mice do not show obvious disease phenotypes in the absence of metabolic stress, and the lifespan of this strain was similar to that of *C57BL*/*6J*. The ratio of Firmicutes to Bacteroidetes was higher in *C57BL*/*6J-mt* ^*FVB*/*NJ*^ compared with *C57BL*/*6J*, indicating a potential association to obesity in *C57BL*/*6J-mt* ^*FVB*/*NJ*^. Body weight of this strain shows no trends towards obesity under normal chow, which suggests a predisposition only under metabolic stress. Our findings indicate that the phenotypic differences observed in this strain in the absence of metabolic stress (i.e. increased glycolysis, less activity, dysbiosis in the gut) are tolerated without major health consequences until death. In contrast, metabolic stimulus such as dietary modification could exceed certain thresholds, resulting in more severe disease phenotypes. In line with this hypothesis, we previously reported that *C57BL*/*6J-mt* ^*FVB*/*NJ*^ mice are more susceptible to experimental metabolic diseases, such as insulin resistance^55^, diet-induced obesity and non-alcoholic steatohepatitis (NASH)^54^. Furthermore, another conplastic strain carrying the same *mt-Atp8* mutation displays increased susceptibility to chronic inflammatory diseases, e.g. collagen-induced arthritis and lupus nephritis^56^ (Supplementary Table S4). Our findings offer a potential mechanistic explanation for these previously reported phenotypes: we hypothesize that the *mt-Apt8* mutation primarily causes a shift in metabolism in the host, which results into the shift in the microbiota and thereby the metabolites in the gut. Alternatively, the shift in the microbiota could be an independent phenomenon of the mutation, which may contribute to the host’s metabolic disequilibrium.

In summary, our data highlight evidence that mutations in mtDNA influence the composition of gut microbiota, primarily by modulating mitochondrial functioning. The *mt-Atp8* mutation in *C57BL*/*6J-mt* ^*FVB*/*NJ*^ mice significantly modulates key cellular metabolic processes, including the level of OXPHOS respiration and glycolysis, consequently changing the metabolite composition of host cells, which appears to lead to a shift of the microbial composition in the gut. The gut microbiota in turn has the potential to regulate gene expression involved in signaling, e.g. immune responses, which may further result in increased susceptibility to common diseases in the context of additional triggers (e.g. immunological or metabolical stimulus) in *C57BL6J-mt* ^*FVB*/*NJ*^.

As shown in this study, the crosstalk between mtDNA/mitochondria and microbiota should be considered as a critical factor to elucidate the pathology and potential novel therapeutic options for common diseases.

## Methods

### Mice

*C57BL*/*6J* mice were obtained from Jackson Laboratory (Bar Harbor, USA) and bred in the animal facility of the University of Lübeck. The conplastic strains *C57BL*/*6J-mt* ^*FVB*/*NJ*^ and *C57BL*/*6J-mt* ^*NZB*/*BlnJ*^ were generated as described previously^27^. Conplastic strains were maintained by repeated backcrossing female conplastic offspring with male *C57BL*/*6J* mice, which were randomly selected from the *C57BL*/*6J* colony maintained in the same breeding facility room. With this breeding strategy there was ample opportunity for microbiota to be homogenized between the strains, i.e. genetic factors are not confounded by separate housing.

The mutations in the mtDNA of each conplastic strains are listed in Supplementary Table S5. Fresh fecal samples were collected from mice at three months of age from nine females and seven males per strain.

Animal use and all methods were approved by the Animal Care and Use Committee (V242-7224. 122-5, Kiel, Germany), and were performed in accordance with the relevant guidelines and regulations by certified personnel.

### DNA extraction and 16S rRNA gene sequencing

DNA was extracted from fecal samples with the PowerSoil Kit (MoBio, Carlsbad, CA) following the manufacturer’s protocol. The 16S rRNA gene was amplified using uniquely barcoded primers flanking the V1 and V2 hypervariable regions (27F-338R) with fused MiSeq adapters in a 25 µl PCR as previously described^28^. The reaction mix consist of one µL of the template DNA, four µL of each forward and reverse primer (0.28 µM), 0.25 µL of Phusion Hot Start II DNA polymerase (0.5 units), 0.5 µL dNTPs (200µM each) and five µL of HF buffer. PCR amplification was performed with the condition described in Supplementary Table S6. The concentration of the PCR products was estimated on 1.5% agarose gels using the image software Quantum Capt v16.04 (Vilber Lourmat Deutschland GmbH, Eberhardzell, Germany) with a known concentration of DNA marker as the internal standard for band intensity measurement. The samples of individual gels were pooled into approximately equimolar subpools, as indicated by band intensity. The concentration of subpools was measured with the Quibit dsDNA BR Assay Kit (Life Technologies GmbH, Darmstadt, Germany), followed by purification with Agencourt AMPure Beads (Beckman Coulter GmbH, Krefeld, Germany) and quantification of library size and concentration using a Bioanalyzer (Agilent Technologies, Waldbronn, Germany). Subpools were mixed in equimolar amounts and stored at −20 °C until sequencing. Sequencing was performed on the Illumina MiSeq platform with v3 chemistry.

### MiSeq data analysis

CASAVA version 1.8.2 was used to demultipex raw sequencing reads. Afterwards, reads were merged using *fastq_mergepairs* from VSEARCH version 1.9.9^57^ with a maximum number of mismatches of 12 and the merged reads (contigs) between 270 and 330 bp long. Next, a quality filter step was applied as implemented in the *fasta_filter* command from the USEARCH package version 8.1.1861^58^ with the expected numbers of errors set to 0.5. Chimeras were identified by *uchime_ref* ^59^ (USEARCH) with the RDP Gold database version 9 as a reference database and were removed from the contig set.

### Taxonomic classification

Taxonomy was assigned to the genus level for all non-chimeric sequences using Mothur version 1.36.1^60^ and the SILVA database version 123^61^ with 80% bootstrap support (1,000 iterations). Sequences of non-bacterial origin were removed from the data set and remaining sequences were aligned to the 16S rRNA V1-V2 region against the SILVA database. Next, 12,500 sequences per individual were chosen randomly to normalize for differences in read number between individuals. Operational taxonomic units (OTUs) were assigned at a 97% similarity threshold and the consensus sequence for each OTU was chosen using a distance-based method. Finally, we used FastTree version 2.1.4^62^ to construct a phylogenetic tree with a generalized time-reversible (GTR) substitution model and the gamma option to rescale branch length. The resulting tree was rooted using the midpoint method for rooting. OTUs present in only one individual with one contig (singletons) were removed from the data prior to further analyses.

### Alpha and beta diversity analysis

Three alpha diversity indices (Chao1, Shannon, Simpson) were estimated using the *vegan* package version 2.3–4^63^. Unweighted UniFrac distance (beta diversity) was estimated using the phyloseq package version 1.14.0^64^. To investigate differences in beta diversity, non-parametric analysis of variance was used as implemented in the *adonis* command (*vegan* package). To compute significance values for each factor, 999 permutations were run. Principle coordinate analysis was performed using the *ordinate* command as implemented in the *phyloseq* package. Constrained analysis of principle coordinates was carried out using *capscale* (*vegan* package) and significance of constraints was assessed with 999 permutations for each factor and for each cap-axis using an anova-like permutation test^65^.

### Indicator species analysis

Indicator species analysis was performed as previously described^66^ using *indicspecies* version 1.7.5 with the specific site group function *IndVal*.*g* and 10,000 permutations to assess significance values. OTUs with an adjusted p-value (Benjamini-Hochberg correction) below 0.05 were considered as significant indicator species.

### Statistical analyses

Unless stated otherwise, all statistical analyses were performed using R Version 3.2.4^67^. When necessary, correction for multiple testing was applied using the Benjamini-Hochberg method^68^ as implemented in the *p*.*adjust* command (*stats* package for R). Throughout the manuscript *Padj* denotes corrected p-values, whereas *P* denotes uncorrected p-values. To test for differences between all three strains, Kruskal-Wallis tests were performed and Dunn tests were applied as *post-hoc* tests with Benjamini-Hochberg correction for multiple testing.

### Phenotyping of *C57BL/6J* and *C57BL/6J-mt*^*FVB/NJ*^ mice

See supplementary materials and methods.

### Data availability

Sequencing data used for this study was submitted to the European Nucleotide Archive (ENA) and is available under accession number PRJEB14844.

## Electronic supplementary material

Supplementary information

Supplementary Table S1, Supplementary Table S2
