# Supplementary material for: Mitochondrial gene polymorphism is associated with gut microbial communities in mice
Source: Sci Rep. 2017 Nov 10;7:15293. doi: 10.1038/s41598-017-15377-7 (PMC5681637; doi:10.1038/s41598-017-15377-7)
Supplement: Supplementary file 1 — Supplementary information [file 41598_2017_15377_MOESM1_ESM.pdf]

Supplementary information

Mitochondrial gene polymorphism is associated with gut microbial communities in mice

Misa Hirose, Axel Künstner, Paul Schilf, Annika Sünderhauf, Jan Rupp, Olaf Jöhren, Markus Schwaninger, Christian Sina, John F Baines, and Saleh M Ibrahim

Figure S1: Basal metabolic phenotype of *C57BL/6J-mt$^{FVB/NJ}$* and *C57BL/6J*.

Figure S2: Lifespan in *C57BL/6J-mt$^{FVB/NJ}$* (FVB) and *C57BL/6J* (B6).

Table S1: Taxonomic analysis at the family level (.xlsx).

Table S2: Indicator species for conplastic strains (.xlsx).

Table S3: Statistical analysis for the lifespan of *C57BL/6J* and *C57BL/6J-mt$^{FVB/NJ}$*.

Table S4: Susceptibilities to experimental diseases in *C57BL/6J-mt$^{FVB/NJ}$*.

Table S5: Mutations in mtDNA in conplastic strains.

Table S6: PCR condition used for 16SrRNA sequencing library preparation.

Supplementary methods

Supplementary references

Figure S1

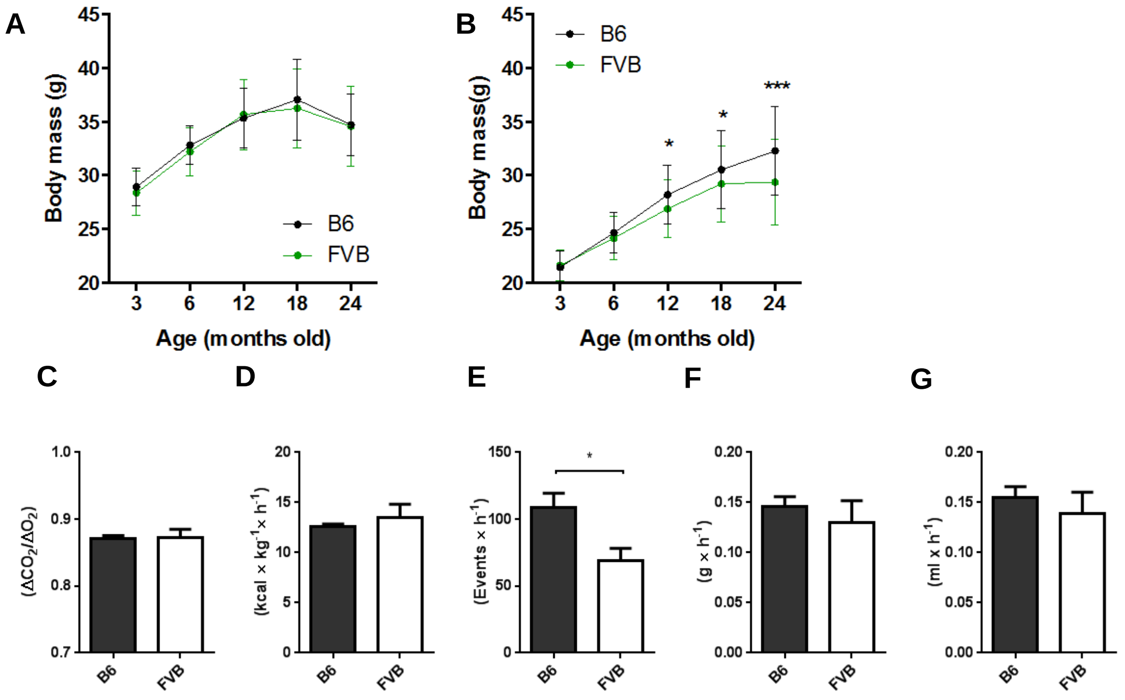

Supplementary Figure S1: Basal metabolic phenotype of *C57BL/6J-mt$^{FVB/NJ}$* and *C57BL/6J*. Body weight at different ages was evaluated in males (**A**) and females (**B**). N≥52/strain/age group. Female mice at the age of 3 months were evaluated for respiratory exchange ratio (**C**), energy expenditure (**D**), locomotor activity (**E**), and food/water (**F**, **G**, respectively) using a indirect calorimety cage system. N=23 (*C57BL/6J*) and n=8 (*C57BL/6J-mt$^{FVB/NJ}$*). B6, *C57BL/6J*; FVB, *C57BL/6J-mt$^{FVB/NJ}$*. *, $P<0.05$, *t* test.

Figure S2

A

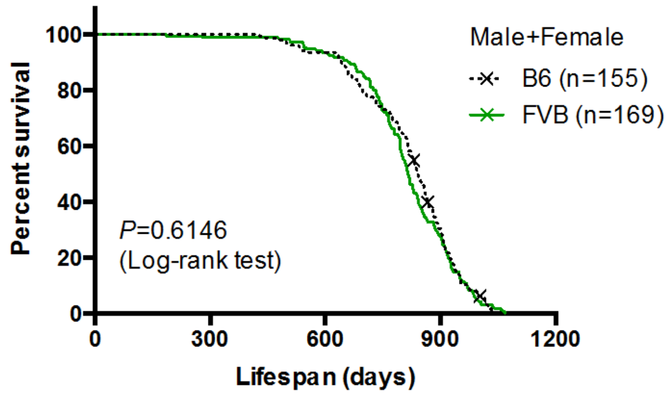

B

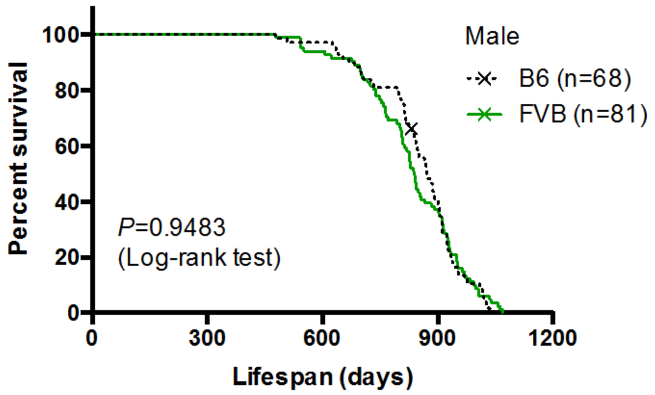

C

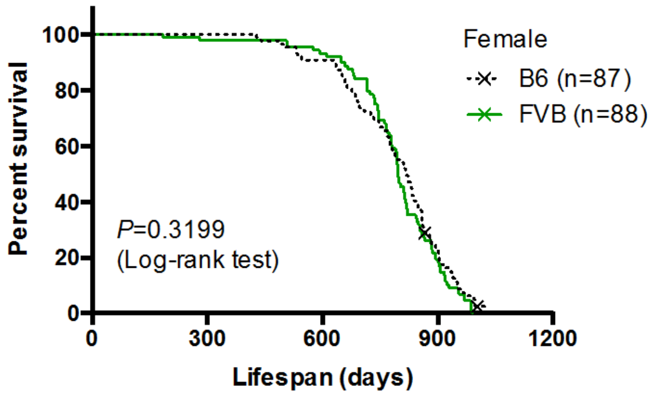

Supplementary Figure S2: Lifespan in *C57BL/6J-mt*$^{FVB/NJ}$ (FVB) and *C57BL/6J* (B6). (A) both sexes, **(B)** male, **(C)** females. A cross (x) indicates a censored animal, which was alive at the time of data collection.

Table S1 and S2: Supplementary Info File (.xlsx)

Table S3: Statistical analysis for the lifespan of *C57BL/6J* and *C57BL/6J-mt*$^{FVB/NJ}$.

Sex	Strain	N	Events	Median (days)	Log-rank	Gehan
Total	*C57BL/6J*	155	151	841.0	0.6146	0.4006
	C57BL/6J-mt$^{FVB/NJ}$	169	169	814.0		
Male	*C57BL/6J*	68	67	871.0	0.9483	0.3592
	C57BL/6J-mt$^{FVB/NJ}$	81	81	839.0		
Female	*C57BL/6J*	87	84	817.0	0.3199	0.7995
	C57BL/6J-mt$^{FVB/NJ}$	88	88	795.5		

Table S4: Susceptibilities to experimental diseases in *C57BL/6J-mt$^{FVB/NJ}$*.

More susceptible in C57BL/6J-mt$^{FVB/NJ}$	**Reference***
Pancreatic beta cell dysfunction	1
Diet-induced obesity	2
Non-alcoholic steatohepatitis (NASH)	2
Lupus nephritis	3
Autoimmune pancreatitis	3
Type 1 diabetes	3
Collagen-induced arthritis	3

*****See **Supplementary references.**

Table S5: Mutations in mtDNA in conplastic strains.

Position	Gene	Mutation	Amino acid	*C57BL/6J*	*C57BL/6J-mt$^{FVB/NJ}$*	*C57BL/6J-mt$^{NZB/BlnJ}$*
7778	*mt-Atp8*	G>T	Asp-Tyr	G	T	G
9461	*mt-Nd3*	T>C	Met-Met	T	C	C
9821	*mt-Tr*	A-ins		8A	9A	10A

Table S6: PCR condition used for 16SrRNA sequencing library preparation.

Step	Temperature (°C)	Time	
1. Initial denaturation	98	30 sec	
2. Denaturation	98	9 sec	
3. Annealing	50	1 min	30 cycles
4. Extension	72	90 sec	
5. Final extension	72	10 min	
6. Hold	12		

Supplementary methods

Mouse phenotyping studies

Mice used in each phenotyping studies were independent.

Body mass

At the age of three, six, 12, 18 and 24 months, body mass of mice were evaluated and recorded.

Indirect calorimetric cage analysis

Oxygen consumption, carbohydrate production, food and water intake, as well as locomotor activity were continuously monitored using an open-circuit indirect calorimetry system (PhenoMaster SystemTM, TSE, Bad Homburg, Germany). Mice were allocated individually to the experimental room system 4 days before measurements start so that they are able to acclimatize, followed by monitoring for 5 days. The average values recorded during the last 3 days of the measurement were used for the analysis. The respiratory exchange ratio (RER) was estimated as ratio of CO_2 produced (ml/h) to O_2 consumed (ml/h). Energy expenditure (EE) was calculated as following: (3.941 + 1.106 X RER) X O_2 consumed and normalized to body weight[4].

Cell metabolism assay in primary lymphocytes

To evaluate metabolic phenotype of primary lymphocytes isolated from mice, oxygen consumption rate (OCR, in pmol/min) and extracellular acidification rate (ECAR, in mpH/min) were measured using the Seahorse XF24 analyzer (Seahorse Bioscience, North Billerica, USA) according to the manufacturer´s protocol. Lymphocytes were resuspended into DMEM, and plated onto a 24-well Seahorse XF-24 assay plate coated with Cell-Tak (BD Bioscience) at 10^6 cells per well, to allow cells to attach the plate. Cells were incubated under 5% CO_2, at 37°C for 1 hour to recover before the assay.

OCR assay. Measurement was started after replacing the medium into XF Mitostress assay buffer (DMEM without bicarbonate supplemented with 4.5g/L D-glucose, 2mM L-glutamine, 1mM pyruvate, pH7.4). After base line measurement, 1µM oligomycin was added to inhibit the ATP synthase, thereby determine ATP-linked oxygen consumption and proton leak). Next, 1µM FCCP was injected to uncouple the mitochondrial membrane potential, allowing maximum electron flux to determine maximal respiration and spare capacity. Lastly, a mixture of 1µM antimycin A and 1µM rotenone was injected to completely inhibit mitochondrial electron transport and define the non-mitochondrial oxygen consumption.

ECAR assay. The measurement was started after replacing the medium with XF glycolysis assay buffer (DMEM without bicarbonate supplemented with 2mM L-glutamine, pH7.4). Basal glycolytic activity was measured by adding 10mM glucose, and the enhanced glycolytic activity under stress condition was accessed by adding 1µM oligomycin, followed by measurement of the glycolysis-independent ECAR values by adding 100mM 2-deoxyglucose.

Lifespan study

To detect a 10% difference in lifespan with a p-value of 0.05 and a power of 0.8, sixty- four mice per group were calculated using G*Power[5]. Therefore, 68 male *C57BL/6J* mice, 87 female *C57BL/6J* mice, 79 male *C57BL/6J-mt*$^{FVB/NJ}$ mice and 88 female *C57BL/6J-mt*$^{FVB/NJ}$ mice were assigned to the longitudinal study. All mice used in the lifespan study had no mating experience. Mice were daily inspected by an experienced animal facility staff and if any unusual observation, including diseases and behavioral changes, it was reported to a veterinarian. All findings were recorded.

Statistical analysis for the animal experiments

GraphPad Prism 6 was used for the statistical analysis. Statistical analysis for the body weight study and indirect calorimetric assay was performed using two-way ANOVA. Results for metabolites were analyzed using t-test. For the lifespan study survival curves were drawn using the Kaplan-Meier method, and median lifespan with 95% confidence intervals were calculated. Censored mice were displayed with a line in the figure. The difference of longevity between the strains was analyzed using both the log-rank method and the Gehan test. P-values less than 0.05 were considered as statistically significant difference.

Supplementary references

1. Weiss H. et al. The mitochondrial Atp8 mutation induces mitochondrial ROS generation, secretory dysfunction, and β-cell mass adaptation in conplastic B6-mtFVB mice. *Endocrinology* **153,** 4666–4676 (2012).
2. Schröder, T. *et al.* Mitochondrial gene polymorphisms alter hepatic cellular energy metabolism and aggravate diet-induced non-alcoholic steatohepatitis. *Mol Metab* **5,** 283–295 (2016).
3. Yu, X. *et al.* The mtDNA nt7778 G/T polymorphism affects autoimmune diseases and reproductive performance in the mouse. *Hum. Mol. Genet.* **18,** 4689–4698 (2009).
4. Weir, J.B.D.B. New methods for calculating metabolic rare with special reference to protein metabolism. *J. Physiol. (Lond.)* **109**, 1-9 (1949).
5. Faul, F., Erdfelder, E., Lang, A-G.& Buchner, A. G*Power 3: a flexible statistical power analysis program for the social, behavioral, and biomedical sciences. *Behav Res Methods* **39**, 175-191 (2007).
